# Relationship Between Left Ventricular Ejection Fraction Variation and Systemic Vascular Resistance: A Prospective Cardiovascular Magnetic Resonance Study

**DOI:** 10.3389/fcvm.2021.803567

**Published:** 2021-12-24

**Authors:** Damien Mandry, Nicolas Girerd, Zohra Lamiral, Olivier Huttin, Laura Filippetti, Emilien Micard, Marine Beaumont, Marie-Paule Bernadette Ncho Mottoh, Nathalie Pace, Faïez Zannad, Patrick Rossignol, Pierre-Yves Marie

**Affiliations:** ^1^CHRU-Nancy, Université de Lorraine, Department of Radiology, Nancy, France; ^2^Université de Lorraine, INSERM, UMR-1254, Nancy, France; ^3^Université de Lorraine, INSERM, UMR-1116, Nancy, France; ^4^CHRU-Nancy, Université de Lorraine, Department of Cardiology, Nancy, France; ^5^Université de Lorraine, CHRU-Nancy, INSERM, CIC 1433, Nancy, France; ^6^FCRIN INI-CRCT, Nancy, France; ^7^CHRU-Nancy, Université de Lorraine, Department of Nuclear Medicine and Nancyclotep, Nancy, France

**Keywords:** flow-encoding sequence, ejection fraction, systemic vascular resistance, hypertension, obesity, cardiovascular magnetic resonance

## Abstract

**Introduction:** This cardiovascular magnetic resonance (CMR) study aims to determine whether changes in systemic vascular resistance (SVR), obtained from CMR flow sequences, might explain the significant long-term changes in left ventricular (LV) ejection fraction (EF) observed in subjects with no cardiac disease history.

**Methods:** Cohort subjects without any known cardiac disease but with high rates of hypertension and obesity, underwent CMR with phase-contrast sequences both at baseline and at a median follow-up of 5.2 years. Longitudinal changes in EF were analyzed for any concomitant changes in blood pressure and vascular function, notably the indexed SVR given by the formula: mean brachial blood pressure / cardiac output x body surface area.

**Results:** A total of 118 subjects (53 ± 12 years, 52% women) were included, 26% had hypertension, and 52% were obese. Eighteen (15%) had significant EF variations between baseline and follow-up (7 increased EF and 11 decreased EF). Longitudinal changes in EF were inversely related to concomitant changes in mean and diastolic blood pressures (*p* = 0.030 and *p* = 0.027, respectively) and much more significantly to SVR (*p* < 0.001). On average, these SVR changes were −8.08 ± 9.21 and +8.14 ± 8.28 mmHg.min.m^2^.L^−1^, respectively, in subjects with significant increases and decreases in EF, and 3.32 ± 7.53 mmHg.min.m^2^.L^−1^ in subjects with a stable EF (overall *p* < 0.001).

**Conclusions:** Significant EF variations are not uncommon during the long-term CMR follow-up of populations with no evident health issues except for uncomplicated hypertension and obesity. However, most of these variations are linked to SVR changes and may therefore be unrelated to any intrinsic change in LV contractility. This underscores the benefits of specifically assessing LV afterload when EF is monitored in populations at risk of vascular dysfunction.

**Clinical Trial Registration:** ClinicalTrials.gov, identifier: NCT01716819 and NCT02430805.

## Introduction

The left ventricular (LV) ejection fraction (EF) remains extensively used to quantify LV systolic performance (1), with cardiovascular magnetic resonance (CMR) imaging being the reference technique for measuring EF and monitoring EF changes (1–4). The major limitation of this type of approach is the EF dependence on loading conditions which is also an issue for most other parameters used to assess LV systolic function (5). An individual's EF measurement is not constant but varies, particularly as a function of the afterload and blood pressure (BP) (6–8). It is therefore generally recommended to record the bracial BP observed during individual EF measurements (9–11).

Nowadays LV afterload is assessed more specifically and non-invasively by combining the information from brachial BP to the stroke volume values provided by CMR flow-velocity sequences (12–14). This approach allows to measure several parameters known to reflect or to greatly impact LV afterload, notably systemic vascular resistance (SVR), effective arterial elastance (Ea), and total arterial compliance (TAC) (12–14).

A previously published CMR study showed that EF and SVR measurements were interdependent in the months following an acute myocardial infarction (MI), with the increase in EF associated with a concomitant decrease in SVR under a post-MI vasodilating medical regimen (13). It is however unclear whether such SVR changes might also explain the significant EF variations observed in subjects with no evident cardiac disease but at increased risk of developing cardiovascular disease, such as hypertensive and/or obese subjects.

The current CMR-based study aims to determine whether longitudinal changes in LV afterload and particularly in SVR, might explain the significant EF changes observed over time in subjects with no evident health issues except for uncomplicated hypertension and obesity.

## Materials and Methods

### Study Populations

Subjects evaluated in the current study did not have any medical history of cardiac disease. Cardiovascular monitoring was performed using the same CMR protocol with subjects pooled from two different cohorts:

(1) The “Role of the Renin-Angiotensin Aldosterone System in the Mechanisms of Transition to Heart Failure in Abdominal Obesity (R2C2-II)” cohort has already been described elsewhere (12, 14, 15). The cohort included middle-aged subjects (40–65 years) with abdominal obesity, no cardiovascular medication and no cardiovascular disease except for stage 1 hypertension. Subjects were invited to participate in a >4-year longitudinal study, which included CMR at baseline and at follow-up (ClinicalTrials.gov NCT01716819).

(2) The “Predisposition and Transition Mechanisms from Arterial Hypertension to Heart Failure (Hypercare)” family-based study included 58 subjects younger than 60 years of age, with uncomplicated hypertension or a history of familial hypertension. This longitudinal study, which included CMR investigations at baseline and at 4 years, has already been described elsewhere (12) (ClinicalTrials.gov NCT02430805).

The main exclusion criteria for both cohorts were: diabetes; inflammatory or infectious disease; renal, hepatic or pulmonary insufficiency; and a history of malignant disease. The local Ethics Committee approved both cohort studies, with all study participants providing their signed informed consent to participate.

### CMR Recording and Analysis

CMRs were performed on a 3-T or 1.5-T magnet (GE Medical Systems, Milwaukee, WI, USA) (12–15). An automated sphygmomanometer (Maglife C, Schiller Medical, Wissembourg, France) was used to measure brachial blood pressure (BP) during the CMR examinations. Averaged values were used for the analyses presented below.

A steady-state free precession pulse sequence and dedicated software (MASS™, Medis, The Netherlands) were used to measure LV end-diastolic volume, end-diastolic mass, and EF in contiguous short-axis. The concentric remodeling (CR) index was defined as LV mass/end-diastolic volume ratio (12–15).

Cardiac output was determined using a velocity-encoded phase-contrast gradient-echo sequence on a slice positioned perpendicularly to the ascending aorta (12–15). Stroke volume (SV) was determined with the “CV flow” software (Medis, The Netherlands), with velocities corrected using an ROI-based method in instances of obvious offset errors.

Values of cardiac output and stroke volume were used to estimate systemic vascular resistance (SVR: mean pressure/cardiac output) (12–19), effective arterial elastance [Ea = 0.9 × systolic BP (mmHg)/stroke volume (mL)] (12, 15–19), and total arterial compliance index [TAC = stroke volume (mmHg)/pulse pressure (mmHg)] (12, 14–17). All these CMR-derived parameters were indexed to body surface area, except for EF and CR. Since none of the study subjects had any medical history of cardiac disease, the central venous pressure was considered normal and thus negligible for the determination of SVR.

As already detailed elsewhere, abnormally high values of SVR, LV mass and concentric remodeling index were defined as the upper limits of the 95% confidence intervals observed in a healthy non-obese middle-aged population investigated using the same CMR methodology (12). A significant EF change was additionally defined as an absolute difference >8% according to a reproducibility study also performed with the same MRI methodology in our center (13).

### Statistical Analyses

Analyses were performed using the commercially available SAS software version 9.4 (SAS Institute Inc. Cary, NC, USA). Continuous variables are expressed as mean and standard deviations (SD) and categorical variables as numbers and percentages (Table 1). Paired comparisons between baseline and follow-up were evaluated using the Wilcoxon sum rank test for continuous variables and the Mc Nemar test for categorical variables. Spearman correlation coefficients and their 95% CI intervals were computed for the baseline-to-follow-up changes in EF and the selected variables listed in Table 2. Univariate and multivariate ascending regression analyses were performed to check Linear model assumptions with *p*-values <0.05 to enter variables and >0.10 to remove variables (Table 3).

**Table 1:** Comparison of the main recorded data between baseline and follow-up.

	**Baseline**	**Follow-up**	***P*-value**
Age (years)	52.5 ± 12.5	58.5 ± 12.3	_____
Female gender	61 (51.7%)	61 (51.7%)	_____
Body weight (kg)	83.8 ± 14.7	86.5 ± 15.0	0.0004
Body mass index (kg.m^−2^)	29.6 ± 4.8	30.7 ± 4.9	<0.0001
Obesity	61 (51.7%)	65 (55.1%)	0.80
Heart rate (bpm)	70.3 ± 11.5	65.9 ± 10.7	0.002
Systolic BP (mmHg)	128.7 ± 18.1	128.7 ± 16.3	0.60
Diastolic BP (mmHg)	74.7 ± 12.3	76.3 ± 10.4	0.045
Mean BP (mmHg)	92.4 ± 13.2	93.4 ± 10.8	0.15
Pulse BP (mmHg)	54.0 ± 12.9	52.4 ± 13.4	0.13
Indexed stroke volume (mL.m^−2^)	43.1 ± 8.9	41.9 ± 8.8	0.44
Cardiac index (L.min^−1^.m^−2^)	3.00 ± 0.66	2.72 ± 0.53	<0.0001
Indexed SVR (mmHg.min.m^2^.L^−1^)	32.1 ± 7.9	35.6 ± 7.6	<0.0001
Abnormal (>40 mmHg.min.m^2^.L^−1^)	17 (14.4 %)	29 (24.6 %)	0.45
Indexed TAC (mL.mmHg^−1^.m^−2^)	0.84 ± 0.23	0.85 ± 0.27	0.65
Indexed Ea (mmHg.mL^−1^.m^2^)	2.79 ± 0.67	2.89 ± 0.76	0.40
Indexed ESV (mL.m^−2^)	28.9 ± 7.5	28.3 ± 7.6	0.15
Indexed EDV (mL.m^−2^)	71.8 ± 12.2	69.3 ± 13.0	0.006
EF (%)	60.0 ± 6.3	59.6 ± 5.3	0.46
Abnormal (<50%)	5 (4.2%)	4 (3.4%)	0.56
Indexed LV mass (g.m^−2^)	50.5 ± 10.5	48.2 ± 9.6	<0.0001
CR index (g.mL^−1^)	0.71 ± 0.15	0.71 ± 0.14	0.19

*BP, blood pressure; CR, concentric remodeling; Ea, effective arterial elastance; EDV, end-diastolic volume; EF, ejection fraction; LV, left ventricle; TAC, total arterial compliance; SVR, systemic vascular resistance*.

**Table 2:** Association between longitudinal changes in LVEF and baseline and concomitant changes in clinical and hemodynamic variables.

**Parameter**	**r_**s**_ (95% CI)**	***P*-value**
Female gender	−0.60 (−2.05, 0.84)	0.72
Body mass index (kg.m^−2^)	−0.01 (−0.19, 0.17)	0.91
Δ from baseline	−0.09 (−0.27, 0.09)	0.31
Age (years)	−0.13 (−0.30, 0.05)	0.16
Δ from baseline	0.02 (−0.16, 0.20)	0.84
Heart rate (bpm)	0.02 (−0.16, 0.20)	0.84
Δ from baseline	−0.10 (−0.28, 0.08)	0.27
Systolic BP (mmHg)	−0.03 (−0.21, 0.16)	0.78
Δ from baseline	−0.08 (−0.26, 0.10)	0.38
Diastolic BP (mmHg)	0.06 (−0.12, 0.24)	0.52
Δ from baseline	−0.20 (−0.37, −0.02)	0.027
Mean BP (mmHg)	0.06 (−0.12, 0.24)	0.50
Δ from baseline	−0.20 (−0.36, −0.02)	0.031
Pulse BP (mmHg)	−0.10 (−0.27, 0.09)	0.30
Δ from baseline	0.10 (−0.08, 0.27)	0.29
Indexed EDV (mL.m^−2^)	0.07 (−0.06, 0.12)	0.484
Δ from baseline	0.05 (−0.16, 0.05)	0.330
Baseline EF	−0.63 (−0.73, −0.50)	<0.0001
Indexed SVR (mmHg.min.m^2^.L^−1^)	0.32 (0.14, 0.47)	0.0005
Δ from baseline	−0.44 (−0.57, −0.28)	<.0001
Indexed TAC (mL.mmHg^−1^.m^−2^)	−0.13 (−0.30, 0.06)	0.18
Δ from baseline	0.19 (0.01, 0.36)	0.038
Indexed Ea (mmHg.mL^−1^.m^2^)	−0.17 (−0.34, 0.01)	0.059
Δ from baseline	−0.42 (−0.56, −0.26)	<0.0001

**Table 3:** Linear regression models obtained through forward selections, with Beta coefficients [standard error (SE)], *P* and *R*^2^ values, for predicting the follow-up to baseline differences in EF (A). The model was additionally built after excluding the baseline EF value (B).

		**Beta (SE)**	***P*-value**	**Global *R*^2^**
A	Intercept	30.83 ± 3.66	<0.0001	
	Change in SVR	−0.22 ± 0.05	<0.0001	
	Baseline EF	−0.51 ± 0.06	<0.0001	0.50
B	Intercept	0.51 ± 0.50	0.31	
	Change in SVR	−0.30 ± 0.06	<0.0001	0.19

## Results

### Baseline Characteristics of the Study Population

The study population consisted of a total of 118 subjects. As detailed in Table 1, the mean age was 53 ± 12 years, 62 (52%) were women, and 61 (52%) were mild to moderately obese with body mass indexes ranging from 30 to 40 kg.m^−2^.

Based on the inclusion criteria, none of these 118 subjects had any history of cardiovascular disease except for uncomplicated hypertension (*n* = 31, 26%). CMR did not detect any significant cardiac abnormalities, except that 5 subjects had an EF below the 50% level (EF ranging from 43 to 47%), 3 and 17 subjects, respectively, had a higher-than-normal LV mass and CR index.

### Evolution at Follow-Up

Follow-up investigations, performed at a median of 5.2 years from baseline (range 3.7–8.4 years), provided evidence of significant increases in body weight, body mass index, diastolic BP and SVR, compared to baseline (Table 1).

Only 4 subjects (3%) had <50% EF at follow-up, but 18 (15%) had significant EF variations between baseline and follow-up (7 increased EF and 11 decreased EF). The 4 patients with <50% EF at follow-up exhibited a significant EF deterioration during follow-up (−7.8 ± 6.0%), compared to the other patients (0.35 ± 6.0%, *p* = 0.01). All were male with no specific differences in baseline characteristics compared to other male study subjects.

Among the 5 subjects with <50% EF at baseline, only one still had <50% EF at follow-up, whereas the 4 others had EF increases that exceeded the 50% level at follow-up.

### Correlates of Baseline-to-Follow-Up Changes in EF

As detailed in Table 2, EF differences between baseline and follow-up were inversely correlated to the corresponding differences in mean BP (*p* = 0.03) and diastolic BP (*p* = 0.027).

This predictive value of BP-changes was however much lower than that provided by the baseline-to-follow-up changes in effective arterial elastance (Ea) and in SVR (both *p* < 0.001; Table 2). The baseline levels of EF, Ea and SVR were additional univariate predictors, whereas all other analyzed parameters were not (Table 2).

**Figure 1:**
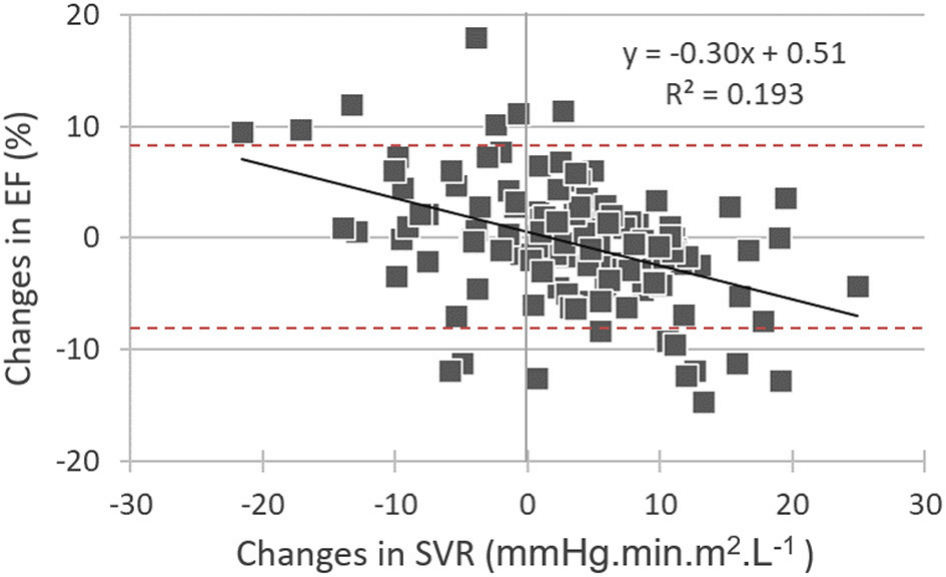
Correlations between baseline-to-follow-up differences in: (i) left ventricular ejection fraction (EF changes) and (ii) systemic vascular resistance (SVR changes). It may additionally be observed that many EF changes are outside of the −8% to +8% interval (red dashed lines) and may thus be considered significant.

On the multivariate regression analysis, which considered all the significant univariate predictors from Table 2, EF changes were significantly and independently related to the baseline-to-follow-up change in SVR together with the baseline EF value (see Table 3). Only the follow-up change in SVR was kept in the model after baseline EF had been excluded (Table 3). Figure 1 displays the inverse association between the follow-up changes in EF and SVR.

**Figure 2:**
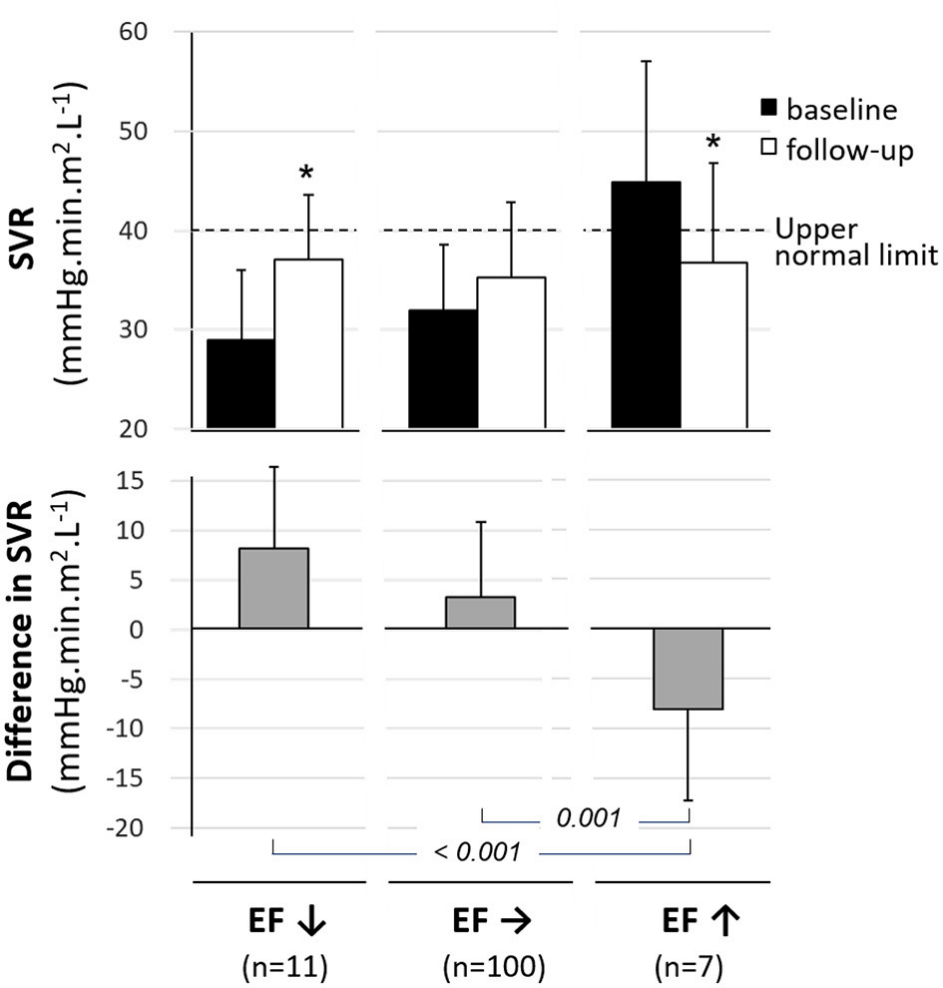
Mean values (±SD) for baseline (black columns) and follow-up (white columns) levels of systemic vascular resistance (SVR, upper panel) and for the mean difference in SVR between baseline and follow-up (gray columns, median panel) in subjects categorized in 3 groups based on baseline-to-follow-up variations in LV ejection fraction -i.e., significant decrease (EF↓), significant increase (EF↑) and stable EF (EF→). *p < 0.05 for paired comparisons between baseline and 6 months.

As illustrated in Figure 2, baseline-to-follow-up changes in SVR were markedly different between subjects with a significant increase in EF at follow-up and those with a significant decrease in EF (−8.08 ± 9.21 mmHg.min.m^2^.L^−1^ vs. 8.14 ± 8.28 mmHg.min.m^2^.L^−1^, *p* < 0.001). The remaining subjects with stable EFs had no significant baseline-to-follow-up changes in SVR (SVR difference in this group: 3.32 ± 7.53 mmHg.min.m^2^.L^−1^) (Figure 2).

Finally, the percentage value of significant EF variations over time (>8%), initially observed in the overall study population (15.3%), was significantly lower after EF-changes had been adjusted for SVR-changes and baseline EF using equations shown in Table 3 (5.9%, *p* = 0.013).

## Discussion

In a population with no history of cardiac disease but including hypertensive and obese subjects, the present CMR study shows that significant long-term EF variations are not uncommon, affecting some 15% of the study population. These variations correlate to SVR changes and may thus be unrelated to any intrinsic changes in LV contractility.

SVR is the main component of the LV afterload and reflects the opposing resistance of the microcirculation that must be overcome by the LV to eject blood. In the current study population, mean SVR increased over time (Table 1), consistent with a functional deterioration of the microcirculation. Such a deterioration is often associated with the aging process, together with an increase in the stiffness of large arteries and may be further promoted by hypertension and obesity (20, 21). Our study population's high rate of hypertension (26%) and obesity (52%) may potentially accelerate the rate of SVR deterioration, consequently impacting the EF. Subjects with isolated obesity have already been shown to exhibit a significant deterioration in large-vessel compliance as well as an increase in the vascular resistance of small resistive vessels, compared to non-obese subjects, using the same CMR protocol (14, 15).

The interdependence of the EF on cardiac loading conditions is well-established. Despite this limitation, EF remains extensively used to quantify LV systolic performance (1). The current recommendation therefore requires brachial BP to be reported for each EF measurement (9–11). The change in BP was a significant predictor of EF change but only for diastolic and mean BP levels. Diastolic BP has already been shown to have a greater impact on EF than systolic BP, particularly in heart failure with preserved EF (6).

We also measured more specific functional arterial parameters from conventional CMR flow sequences. These allowed to determine aortic stroke volume, independently of other CMR sequences used to assess LV function, and more accurately than Doppler-based techniques (22). Combining these stroke volume values with brachial BP measurements allowed to evaluate three functional vascular parameters: (i) total arterial compliance (TAC) index, which is predominantly determined by the great elastic arteries (12, 14–17) (ii) systemic vascular resistance (SVR), which is mainly attributed to the resistive microvessels (12–19), and (iii) global arterial load (Ea), a comprehensive measure of the arterial load that depends on both arterial compliance and arterial resistance (12, 15–19) and is strongly linked to LV remodeling (12, 15). However, Ea-changes were not found to be better predictors of EF variations than SVR-changes in the current study. This is consistent with what was previously reported in post-myocardial infarction patients (13). SVR changes presumably impact stroke volume and EF more directly, than Ea changes -i.e., small arteries which contribute to SVR not only constitute a main component of the LV afterload but also the exit door through which the stroke volume needs to pass before leaving the arterial tree.

A significant decrease in SVR has already been shown to be the predominant mechanism by which the EF increases during exercise in heart disease patients (23), and during the months following a myocardial infarction (13). A vasodilator-related decrease in SVR was additionally shown to be associated with proportional increases in stroke volume and cardiac output of heart failure patients (24). Additional data from several individual cases included in our cohort also confirm significant variations in cardiovascular function and remodeling after changes in antihypertensive treatment (results not shown). These changes were unfortunately not systematically recorded during the long-term follow-up of this cohort and therefore constitute one of the limitations of the current study. Another limitation is that intrinsic contractility was not directly assessed in this cohort.

It may additionally be pointed out that the EF variations were unrelated to the concomitant changes in LV end-diastolic volume (Table 2), an indicator of the LV preload, contrary to what was documented for SVR, an indicator of LV afterload.

**Figure 3:**
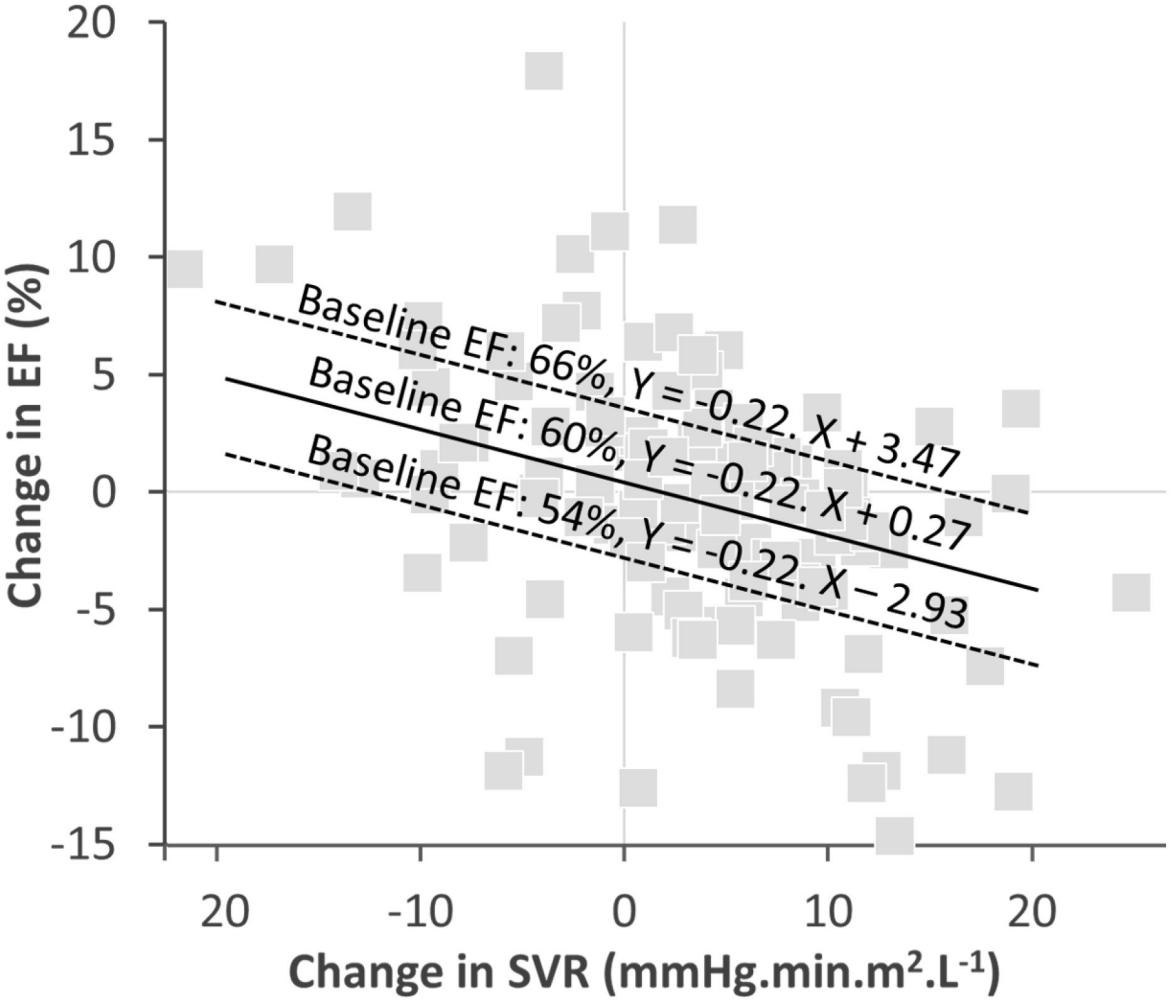
Graph of the correlation between EF-changes and SVR-changes with the regressions computed with the equation from Table 3 for three baseline EF levels: close to the mean (60%), one SD above the mean (66%), and one SD below the mean (54%). The slope of EF- and SVR-changes for the 3 baseline FE levels are identical. However, the intercept corresponding to an absence of any SVR variation are different, with a predicted absolute decrease in EF of ~3% for the 66% EF baseline, an increase of about 3% for the 54% EF baseline and an insignificant predicted change for the 60% EF baseline.

It is also worth mentioning that in addition to the SVR changes, the baseline EF level was an independent predictor of EF variations over time. The impact of baseline EF could at least partly be attributed to a regression to the mean - i.e., a statistic phenomenon that implies that a sample point of a random variable, which is distant from the mean value on a first measurement, has a high probability of being closer to the mean value on a second measurement. This point is further detailed and illustrated in Figure 3.

The current study defined a significant EF variation using an absolute threshold of 8% which corresponds to the results of a reproducibility analysis previously reported by our team using the same CMR methodology (13). The threshold may vary depending on the different conditions tested in the reproducibility analysis (25). This does however not modify the consideration that the rate of EF changes over time may be significantly lowered after adjusting for concomitant SVR changes observed with CMR. Such an adjustment would help identify cases where a decrease in EF relates to vascular rather than myocardial deterioration and ultimately identify different therapeutic targets.

## Conclusion

The current longitudinal CMR study of a cohort including hypertensive and obese subjects shows that significant long-term variations in EF are not uncommon, but that most of these variations are potentially driven by changes in SVR rather than changes in LV contractility. Although a causative relationship is only suggested and remains to be proven, this observation underscores the benefits of specifically assessing LV afterload when EF is monitored in populations at risk of vascular dysfunction.

## Data Availability Statement

The raw data supporting the conclusions of this article will be made available by the authors, without undue reservation.

## Ethics Statement

The studies involving human participants were reviewed and approved by Comité de Protection des Personnes se prêtant à la recherche biomédicale (CPP) - Est. The patients/participants provided their written informed consent to participate in this study.

## Author Contributions

DM, NG, ZL, and P-YM: analysis and interpretation of the data. DM, NG, MB, FZ, PR, and P-YM: writing or revision of the manuscript. OH, LF, EM, M-PN, and NP: study implementation and management of the included subjects. All authors contributed to the article and approved the submitted version.

## Funding

This study was funded by a National Health Ministry (Programme Hospitalier de Recherche Clinique) and the 6th framework program of the European Commission (Ingenious HyperCare Network of Excellence; contract number LSHM-CT-2006-037093).

## Conflict of Interest

The authors declare that the research was conducted in the absence of any commercial or financial relationships that could be construed as a potential conflict of interest.

## Publisher's Note

All claims expressed in this article are solely those of the authors and do not necessarily represent those of their affiliated organizations, or those of the publisher, the editors and the reviewers. Any product that may be evaluated in this article, or claim that may be made by its manufacturer, is not guaranteed or endorsed by the publisher.
